# Compliance with national recommendations for exercise during early pregnancy in a Danish cohort

**DOI:** 10.1186/s12884-015-0756-0

**Published:** 2015-11-27

**Authors:** Lotte Broberg, Anne S. Ersbøll, Mette G. Backhausen, Peter Damm, Ann Tabor, Hanne K Hegaard

**Affiliations:** Department of Obstetrics, Copenhagen University Hospital Rigshospitalet, Blegdamsvej 9, 2100 Copenhagen, Denmark; The Research Unit Women’s and Children’s Health, the Juliane Marie Centre for Women, Children and Reproduction, Copenhagen University Hospital Rigshospitalet, Copenhagen, Denmark; Department of Gynaecology and Obstetrics, Roskilde University Hospital, Roskilde, Denmark; Institute of Clinical Medicine, Faculty of Health and Medical Sciences, University of Copenhagen, Copenhagen, Denmark; Centre for Fetal Medicine, Department of Obstetrics, Copenhagen University Hospital Rigshospitalet, Copenhagen, Denmark; Department of Health Sciences, Child, Family, and Reproductive Health, Faculty of Medicine, Lund University, Lund, Sweden

**Keywords:** Exercise, Recommendation, Preconception, Pregnancy, Antenatal care, Predictors

## Abstract

**Background:**

Exercise during pregnancy is associated with health benefits for both the mother and the fetus, and is therefore recommended in several national guidelines. Only few studies investigate whether these guideline recommendations are met. The aims of this study were 1. To assess the prevalence of pregnant women meeting the Danish recommendations for exercise during early pregnancy, 2. To identify pre-pregnancy factors associated with a lower probability for meeting the recommendations, and 3. To describe which types of exercise pregnant women prefer before and during pregnancy.

**Methods:**

We conducted a cross-sectional study based on a questionnaire during the first trimester among 7,915 women participating in the prospective Copenhagen Pregnancy Cohort. Associations were estimated by multivariate regression analyses.

**Results:**

In early pregnancy, 38 % of the study population met the recommendation for exercise from the Danish Health and Medicines Authority (≥3.5 hours a week).

Multiparity, previous miscarriage use of assisted reproductive technology, no engagement in exercise before pregnancy, smoking, pregnancy following assisted reproductive technology, overweight, not understanding Danish language and a low educational level were all factors associated a lower probability for meeting the recommendations. The preferred types of exercise before and during pregnancy were bicycling, brisk walking, running and strength training. The proportion of women engaged in any type of exercise decreased in early pregnancy with the exception of swimming and aquatic exercise.

**Conclusions:**

In this cohort, more than one-third met the Danish recommendation for exercise during early pregnancy. Exercise in pregnancy is still an issue to address because the most vulnerable groups of pregnant women do not exercise. This is a cause of concern because it may reflect social inequalities in health and highlights the need for a structural and systematic approach to preconception care and early antenatal counselling.

## Background

Exercise is in general associated with psychological well-being and a reduced risk of morbidity and mortality in non-pregnant women [1, 2]. Several studies have documented, that exercise before and during pregnancy is associated with health benefits for both the mother and the unborn child. Exercise before pregnancy is associated with a lower risk of preeclampsia [3] and gestational diabetes mellitus [4]. During pregnancy, exercise is associated with: a lower risk of pregnancy and birth-related complications such as preeclampsia, gestational diabetes mellitus, low back pain, preterm delivery, depression symptoms and emergency caesarean section [3–6].

The physical and mental health benefits of exercise in relation to pregnancy led to a revision of the American College of Obstetrician and Gynecologists’ (ACOG) recommendation concerning exercise and pregnancy in 2002 [7]. Women with a normal pregnancy are recommended to engage in at least 30 minutes of moderate exercise on most if not all days of the week. Likewise, Denmark, Norway and the United Kingdom also published new national recommendations concerning exercise and pregnancy [8–10]. The Danish Health and Medicines Authority recommend at least 30 minutes of moderate intensity exercise daily during normal, uncomplicated pregnancy [9].

Few studies have examined whether pregnant women meet the recommendations concerning exercise during pregnancy more than 10 years after the publication of new exercise recommendations [11–15]. These studies from different countries have found that the majority of women do not meet the recommendations. We seek to promote exercise and prevent inactivity among pregnant women. Thus, it is important to understand whether pregnant women meet the recommendations and to describe the characteristics of pregnant women who do not meet the exercise recommendations.

In addition, it is interesting to examine which types of exercise pregnant women engage in, because information about preferred and well-tolerated types of exercise can be used to target antenatal counselling.

The aims of this study are: 1. To assess the prevalence of pregnant women meeting the Danish recommendations for exercise during early pregnancy, 2. To identify pre-pregnancy factors associated with a lower probability for meeting the recommendations, and 3. To describe which types of exercise pregnant women prefer before and during pregnancy.

## Methods

The data used in this study were from the Copenhagen Pregnancy Cohort, which is a prospective cohort of pregnant women attending the Department of Obstetrics, Copenhagen University Hospital Rigshospitalet, Denmark. The hospital serves as a primary birth facility for women of Copenhagen city and is also a tertiary referral centre with 6,257 deliveries in 2013.

All pregnant women who scheduled an appointment for their first trimester nuchal translucency scan from September 16, 2012 to May 27, 2014 were eligible for this study. In Denmark, pregnant women are offered the first pregnancy consultation with their general practitioner. Subsequently all pregnant women are offered a referral to a nuchal translucency scan as part of the national prenatal screening program, and more than 90 % attend. After the women scheduled an appointment for the nuchal translucency scan, they received an email with a link to a clinical questionnaire available in both Danish and English languages. The women responded at approximately 10 gestational weeks. This questionnaire provides information on reproductive and obstetric history (degree of pregnancy planning, parity, previous miscarriage and method of conception), lifestyle factors before and during the current pregnancy (smoking, exercise, alcohol intake and pre-pregnancy body mass index (BMI)), comorbidities and socio-demographic characteristics (age, education, occupation, cohabiting and Danish language skills). The information was routinely transferred to the pregnant women´s medical records and to a database for research use.

There were 10,250 women who received a link to the clinical questionnaire. After excluding women who were referred to at another hospital for antenatal care and delivery (n = 126), there were 9,649 women eligible of whom 8,650 (89.6 %) completed the questionnaire. Women who miscarried before responding to the questionnaire (n = 475) were not a part of this study population.

We excluded 735 women who did not respond to the questions about exercise before and during pregnancy. The final study population thus consisted of 7,915 (82 %) pregnant women.

### Assessment of exercise

Questions on exercise were phrased as follows:

1. *Did you engage in any exercise the last three months before you became pregnant?* (yes/no)
2. *Do you engage in any exercise at present?* (yes/no)

A positive answer to each of these questions released a question about specific types of exercise and duration (hours/week). The 14 specific types of exercise were as follows: running, strength training, yoga, bicycling (also to and from work), brisk walking, spinning, fitness, swimming, aquatic exercise, horseback riding, racket games, ball games, dance and “other type of exercise”. When marked “other types of exercise” it was possible to write which type/types in free text.

### Definitions of national recommendations for exercise during pregnancy

Women meeting the national recommendation from the Danish Health and Medicines Authority [9] were defined as performing ≥3.5 hours of exercise a week.

### Statistical analysis

Descriptive statistics were used to report characteristics of the study population, and data are presented as numbers and percentages.

Univariate and multivariate logistic regression analyses were performed to assess the possible associations between the dichotomized dependent variable “Meeting the Danish recommendation” (exercising ≥3.5 hours/week versus < 3.5 hours/week) and reproductive and obstetric history, lifestyle factors, health status and socio-demographic characteristics. We included all potential associated predictors categorised and listed in Table 1 in the analyses because the variables were described as confounders in other studies  [16–19] or because they could be considered as potential confounders. The variables in the multivariate logistic regression model were analysed in a multiple stepwise backwards elimination model with stepwise removal of the most insignificant variables that had p > 0.05.

**Table 1:** Characteristics of the study population, n = 7,915

Maternal age (years)	n/(%)*
<25	394 (5.0)
25-29	2342 (29.6)
30-34	3160 (39.9)
35-39	1650 (20.8)
≥40	350 (4.4)
Parity	
Nulliparous	4717 (59.6)
Multiparous	3157 (39.9)
Degree of pregnancy planning	
Very planned	3506 (44.3)
Rather planned	2135 (27.0)
Neither planned nor unplanned	1270 (16.0)
Rather unplanned	354 (4.5)
Very unplanned	313 (4.0)
Method of conception	
Spontaneous	6656 (84.1)
Assisted reproductive technology	894 (11.3)
Smoking before pregnancy	
Yes	758 (9.6)
No	6712 (84.8)
Physical exercise before pregnancy	
Yes	5675 (71.7)
No	2240 (28.3)
Body mass index (kg/m^2^)	
<18.5	409 (5.2)
18.5-24.9	5610 (70.9)
25-29.9	942 (11.9)
≥30	
Disorders	
Chronic disorders*	948 (12.0)
Mental disorders	189 (2.4)
None	5725 (72.3)
Other disorders¤	558 (7.0)
Reads and understands Danish language	
Yes	7505 (94.8)
No	349 (4.4)
Educational level	
Advanced degree	3561 (45.0)
3-4 years higher education	2267 (28.6)
1-2 years higher education	511 (6.5)
Skilled worker	251 (3.2)
No education	524 (6.6)
Occupation	
Employed	5297 (66.9)
Unemployed	469 (5.9)
Student	1182 (14.9)
Other†	275 (3.5)

*Chronic diseases; Hypertension, cardiac diseases, pulmonary diseases, diabetes, metabolic disorders, arthritis, epilepsy and migraine.

¤Other diseases reported were e.g. skin diseases, systemic lupus erythematosis, celiac disease, inflammatory bowel disease, hepatitis, gallstones, multiple sclerosis, thrombophilia, and polycystic ovarian syndrome.

†Including stay at home mothers, pensioners and women on maternity leave.

Due to missing data in some categories will the sum of women differ between categories.

The odds ratios (OR) are presented with 95 % confidence intervals (CI). Statistical significance was defined as a two-sided P-value of <0.05. All statistical analyses were performed in SPSS 19.0 statistical software (IBM).

### Ethical considerations

This study was approved by the Danish Data Protection Agency (no. 2007-58-0015). According to Danish law ethical approval cannot be sought for registry studies. Informed consent was not obtained. The study is a quality improvement project which, according to the Danish Health Authority recommendations valid at the time of implementation, did not require informed consent.

## Results

### Characterisation of the study population

The characteristics of the study population (n = 7,915) are presented in Table 1.

The mean age was 32 years (SD 4.5), and the majority of women were nulliparous, non-smoking, healthy, having 3–4 years of higher education or an advanced degree, employed, living with a partner and had planned the current pregnancy.

Women, who do not respond the question about exercise, were more likely to be nulliparous (68 % vs.60 %), read and understand Danish (98.1 % vs.95.6 %). No differences were seen in maternal age, pre-pregnancy BMI, employment status, cohabiting and education level.

### Compliance with national recommendations

During early pregnancy, 38 % of the women met the recommendation from the Danish Health and Medicines Authority.

### Predictors for decreased probability of meeting the Danish recommendation

The results of the univariate and multivariate logistic regression analyses are presented in Table 2. After mutual adjustment for the variables in the multivariable logistic regression analysis, not engaging in exercise before the present pregnancy (aOR: 0.06; CI 0.05-0.08) was significantly associated with lower probability of meeting recommendations for exercise during pregnancy. Being a multiparous women (aOR 0.73; CI 0.65-0.82), having a previous miscarriage (aOR 0.84; CI 0.74-0.96), and smoking before pregnancy (aOR 0.78; CI 0.63-0.95) were all significantly associated with a lower probability of meeting recommendations for exercise during pregnancy. Additionally, being pregnant following assisted reproductive technology was significantly associated with lower probability of meeting recommendations (aOR 0.76; CI 0.64-0.91). Overweight women with BMI 25–29.9 kg/m^2^ were less likely to meet recommendations (aOR 0.77; CI 0.65-0.92) than women with normal BMI. Women who did not read and understand Danish language were also less likely to meet the recommendations (aOR 0.51; CI: 0.34-0.71), than women who did understand Danish language. Women with no education (aOR 0.69; CI 0.61-0.79), skilled workers (aOR 0.50; CI 0.39-0.65) and women with less than 4 years of higher education (aOR 0.64; CI 0.43-0.87) had a significantly lower probability of meeting recommendations than women with an advanced degree.

**Table 2:** Proportion of pregnant women who met the recommendations and associations with socio-demographic characteristics, obstetric history, lifestyle factors, medical history in the study population (n = 7,915)

	Met Danish recommendation			
	≥3.5 hours/week			
	Yes	No	Crude OR, (95 % CI)	Adjusted* OR, 95 % CI
Age (years)				
<25	107 (27.2)	287 (72.8)	0.57 (0.45-0.72)	
25-29	962 (41.1)	1380 (58.9)	1.062 (0.95-1.19)	
30-34	1252 (39.6)	1908 (60.4)	Ref.	
35-39	584 (35.4)	1066 (64.6)	0.84 (0.74-0.95)	
≥40	107 (47.3)	119 (52.7)	0.67 (0.53-0.85)	
Parity				
Nulliparous	1993 (42.3)	2724 (57.7)	Ref.	Ref.
Multiparous	1016 (32.2)	2141 (67.8)	0.65 (0.59-0.71)	0.73 (0.65-0.82)
Previous miscarriage				
No	2331 (39.7)	3540 (60.3)	Ref.	Ref.
Yes	688 (33.7)	1356 (66.3)	0.77 (0.69-0.86)	0.84 (0.74-0.96)
Degree of pregnancy planning				_
Very planned	1353 (38.6)	2153 (61.4)	Ref.	
Rather planned	897 (42.0)	1238 (58.0)	1.15 (1.03-1.29)	
Neither planned or unplanned	437 (34.4)	833 (65.6)	0.84 (0.73-0.96)	
Rather unplanned	116 (32.8)	238 (67.2)	0.78 (0.62-0.98)	
Very unplanned	95 (30.4)	218 (69.6)	0.69 (0.54-0.89)	
Method of conception				
Spontaneous	2563 (38.5)	4093 (61.5)	Ref.	Ref.
Assisted reproductive technology	319 (35.7)	575 (64.3)	0.89 (0.77-1.03)	0.76 (0.64-0.91)
Smoking before pregnancy				
Yes	217 (28.6)	541 (71.4)	0.61 (0.52-0.72)	0.78 (0.63-0.95)
No	2658 (39.6)	4054 (60.4)	Ref.	Ref.
Physical exercise before pregnancy				
Yes	2658 (48.8)	2784 (51.2)	Ref.	Ref.
No	217 (9.3)	2112 (90.7)	0.06	0.06 (0.05-0.08)
Body mass index (kg/m^2^)				
<18.5	153 (37.4)	256 (62.6)	0.87 (0.71-1.07)	1.00 (0.77-1.28)
18.5-24.9	2289 (40.8)	3321 (59.2)	Ref.	Ref.
25-29.9	289 (30.7)	653 (69.3)	0.64 (0.55-0.75)	0.77 (0.65-0.92)
≥30	98 (30.1)	228 (69.9)	0.62 (0.49-0.80)	1.13 (0.83-1.52)
Disorders				_
Chronic disorders	337 (35.5)	611 (64.5)	0.85 (0.74-0.98)	
Mental disorders	59 (31.2)	130 (68.8)	0.70 (0.51-0.95)	
None	2256 (39.4)	3469 (60.6)	Ref.	
Other disorders	196 (35.1)	362 (64.9)	0.83 (0.69-1.00)	
Reads and understands Danish language				
Yes	2925 (39.0)	4580 (61.0)	Ref.	Ref.
No	72 (20.6)	277 (79.4)	0.41 (0.31-0.53)	0.51 (0.34-0.71)
Cohabiting				_
Yes	2817 (38.9)	4425 (61.1)	Ref.	
No	186 (31.5)	405 (68.5)	0.72 (0.60-0.86)	
Education level				
Advanced degree	1624 (45.6)	1937 (54.4)	Ref.	Ref.
3-4 years higher education	804 (35.5)	1463 (64.5)	0.66 (0.59-0.73)	0.64 (0.43-0.87)
1-2 years higher education	122 (23.9)	389 (76.1)	0.37 (0.30-0.46)	0.58 (0.45-0.74)
Skilled worker	64 (25.5)	187 (74.5)	0.41 (0.31-0.55)	0.50 (0.39-0.65)
No education	137 (26.1)	387 (73.9)	0.42 (0.34-0.52)	0.69 (0.61-0.79)
Occupation				_
Employed	2118 (40.0)	3179 (60.0)	Ref.	
Unemployed	156 (33.3)	313 (66.7)	0.75 (0.61-0.91)	
Student	456 (38.6)	726 (61.4)	0.94 (0.83-1.07)	
Other	55 (20.0)	220 (80.0)	0.38 (0.28-0.51)	

*Adjusted for parity, previous miscarriage, method of conception, smoking before pregnancy, physical exercise before pregnancy, body mass index, cohabiting, education level and occupation

### Types of exercise

**Fig. 1:**
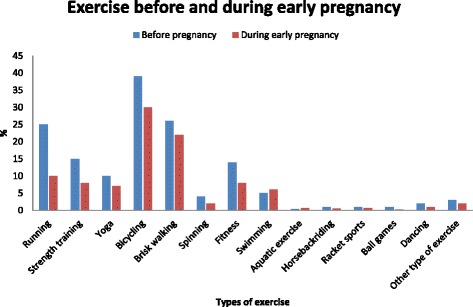
Types of exercise before and during early pregnancy

The four preferred types of exercise before pregnancy were bicycling (39 %), brisk walking (26 %), running (25 %) and strength training (8 %). During early pregnancy bicycling (30 %), brisk walking (22 %), running (10 %) and strength training (8 %) were still the four preferred types of exercise. However, the proportion of pregnant women who were engaged in running was notably reduced. The proportion of women who engaged in all other types of exercise decreased as well except for the proportion of women who engaged in swimming and aquatic exercise. Before pregnancy 5 % and 0.4 % of the women engaged in swimming and aquatic exercise respectively compared to 6 % and 0.6 % in early pregnancy (Fig. 1).

## Discussion

In this population-based study of 7,915 pregnant Danish women in early pregnancy 38 % met the Danish recommendation for exercise. No engagement in exercise before pregnancy, multiparity, previous miscarriage use of assisted reproductive technology, no engagement in exercise before pregnancy, smoking, pregnancy following assisted reproductive technology, overweight, not understanding Danish language and a low educational level were all factors associated with a lower probability for meeting the recommendation.

The compliance with national recommendations for exercise during pregnancy in our study is high compared to other studies. A 2010 Spanish study (n = 1,175) found that 20 % of all pregnant women met the ACOG´s recommended level of exercise in the 20^th^ week of gestation [11]. Another small Spanish study (n = 133) from 2014 found that 5 % of all participants met the ACOG recommendations in both the first and second trimester [14]. Two small studies from Ireland, 2009–2010 (n = 358) and the United States of America, 2006, (n = 311) found that 22 % and 29 %, respectively, met the ACOG recommendations [13, 15]. A 2013 Norwegian study (n = 3,482) found that 15 % of the participating pregnant women, met the national Norwegian recommendation (>3 times a week, >20 minutes at moderate intensity) in gestational weeks 17–21 [12]. The differences in the proportions of women meeting the recommendations might be explained by differences in the study populations, culture or the time of assessment of exercise during pregnancy. The primary outcome was defined as engagement in exercise ≥3.5 hours a week, which is a practical approximation to the Danish Health and Medicines Authority recommendation of 30 minutes of exercise daily [9]. Other studies of pregnancy and exercise also report exercise as hours per week  [11, 13, 14, 16].

A noteworthy proportion of our study population was engaged in exercise before pregnancy, and the majority of the women were highly educated and nulliparous. These factors are associated with exercise during pregnancy [19]. In addition, citizens of Copenhagen are more likely to transport themselves by bicycle. An average citizen in the capital region transport herself 2.4 km by bicycle per day [20], and this may add to the proportion of exercising pregnant women. We assessed exercise at 10 weeks gestation, which was earlier than in other studies [11, 12, 14] and this could explain the higher proportion of pregnant women exercising in our study, as exercise often decreases throughout pregnancy  [21]. Furthermore although the vast majority of the pregnant women at our center is from the local catchment area of Copenhagen the function as a tertiary hospital also providing care to women with high risk pregnancies might influence the exercise patterns.

A Danish study  [19] (n = 88,200) conducted in 1996–2002 before the most recent recommendation for exercise in pregnancy, showed that 7 % of the study population engaged in exercise of ≥ 3.5 hours a week in early/mid pregnancy. Although these are two different study populations, there is a notable increase in the proportion of Danish pregnant women meeting the exercise recommendation. Especially the proportion of pregnant runners has increased. This result may be due to an increased awareness of the importance of exercise and a general belief that it is safe to engage in exercise during pregnancy.

Our study found that conception following assisted reproductive technology and a prior miscarriage were associated with a lower probability of meeting the recommendation for exercise during pregnancy. This emphasises that some groups of pregnant women require special attention regarding exercise and pregnancy. This finding has also been observed in other studies and may be due to concerns about safety [22, 23]. It is reasonable to believe that these women are particularly motivated to have a healthy lifestyle, and as women having assisted reproductive technology are already in contact with the health care system there is an opportunity to provide these women with special preconception care regarding physical activity and exercise.

We found that women with a previous miscarriage had a decreased risk of meeting the recommendation for exercise. This has also been reported in a study from the United States [18]. Another study reported that women with previous miscarriage were afraid that exercise might lead to another miscarriage [22]. Other studies report conflicting results regarding the association between exercise and miscarriage [24–26], but this has not changed the recommendations in neither the United States nor in Europe [9].

Women who do not understand Danish language were less likely to meet the recommendation for exercise. This is in accordance with existing literature [17] and could be explained by differences between beliefs about exercise and women's perceptions of the safety of physical activity during pregnancy in the different ethnic groups [13, 27].

Overweight and physical inactivity both increase the risk of preeclampsia, gestational diabetes mellitus and caesarean section [3, 4, 6, 28–30]. Thus, it is concerning that women with a pre-pregnancy BMI of 25–29.9 kg/m^2^ were less likely to meet the recommendation in our study. It is important for these overweight pregnant women to meet the recommended exercise guidelines during pregnancy to prevent the above-mentioned complications. We did not find a BMI of ≥ 30 kg/m^2^ to be associated with a lesser likelihood to meet the recommendations. This could be explained by the relatively small proportion of obese women in the study population.

Strengths of this study are the large sample size relative to other studies that examined compliance with recommendations for exercise in pregnancy and the high response rate (82 %). Data were collected during early pregnancy with a short recall period, which limits the risk of recall bias. Information about exercise was collected before the women knew anything about the evolution of pregnancy. This minimizes the risk of bias as information collected retrospectively might have been influenced by the evolution of the pregnancy in terms of pregnancy complications, fetal malformations, risk of Down ’s syndrome and other chromosomal abnormalities.

In contrast to other studies  [12, 14, 16, 21, 31], we included both native and English-speaking women, which increases the application of the results to the entire population including other ethnicities. It would have increased the study´s utility further if the questionnaire was translated into more languages than English. Some limitations must be considered. We only report whether the pregnant women meet the recommendation in the first trimester, and we do not have information about exercise throughout pregnancy. Previous studies have shown that pregnant women often reduce physical activity during pregnancy [32]. The questions about exercise are not validated, it would have been ideal to validate the questions with objective measurements of exercise. Nonetheless, the same questions on exercise have been used in The Danish National Birth Cohort as well as used to evaluate the impact of physical activity on the risk of various outcomes  [16, 26]. We asked about the type of exercise and duration (hours/week), but we did not ask about intensity or frequency. These additional questions would have increased the precision and accuracy of the data with respect to whether the pregnant women meet the recommendation of engaging in exercise of moderate intensity.

Approximately 5 % did not attend the nuchal translucency scan and therefore did not receive the questionnaire. If their non-attendance was due to a lower level of education and because they did not read and understand Danish language, it would be expected that fewer of these women met national recommendation for exercise  [17, 19]. We do not know, whether the group attending the nuchal translucency scan differs from that who did not.

It is relevant to emphasize that this study population included well-educated, urban women and that the results may thus not be fully representative of all women in Denmark.

Our data suggest that there are social inequalities in lifestyle factors among pregnant women and highlights the need for structural changes in our preconception and antenatal counselling. According to a Norwegian study, inactive pregnant women are highly motivated and show readiness or intention to increase their physical activity level [33].

## Conclusions

In conclusion, over one-third of pregnant women in this population met the Danish recommendation for exercise in early pregnancy. Exercise among pregnant women is still an important issue to address because the most vulnerable groups of pregnant women do not meet the recommendation. This result is concerning because it may reflect social inequalities in health and highlights the need for a structural and systematic approach to preconception care and early antenatal counselling. It would be useful to assess pregnant women´s views on exercise in pregnancy for insight into the level of awareness among women of the value of exercise for their pregnancy. This would be important in structuring intervention – education vs access campaigns, and research into this is recommended.

## Abbreviations

ACOG: American College of Obstetrician and Gynecologists
BMI: body mass index
